# Comparison of CareStart™ HRP2/pLDH COMBO rapid malaria test with light microscopy in north-west Ethiopia

**DOI:** 10.1186/1475-2875-11-234

**Published:** 2012-07-20

**Authors:** Beyene Moges, Bemnet Amare, Yeshambel Belyhun, Zinaye Tekeste, Muchiye Gizachew, Meseret Workineh, Amare Gebrehiwot, Desalegn Woldeyohannes, Andargachew Mulu, Afework Kassu

**Affiliations:** 1 Department of Immunology and Molecular Biology, School of Biomedical and Laboratory Sciences, College of Medicine and Health Sciences, University of Gondar, 196, Gondar, Ethiopia; 2 Department of Biochemistry College of Medicine and Health Sciences, University of Gondar, Gondar, Ethiopia; 3 Department of Parasitology, School of Biomedical and Laboratory Sciences, College of Medicine and Health Sciences, University of Gondar, Gondar, Ethiopia; 4 Department of Microbiology, School of Biomedical and Laboratory Sciences, College of Medicine and Health Sciences, University of Gondar, Gondar, Ethiopia; 5 Institute of Virology, Leipzig University, Leipzig, Germany

**Keywords:** CareStart™, Light microscopy, Malaria, Rapid diagnostic test, Ethiopia

## Abstract

**Background:**

In Ethiopia, light microscopy is the gold standard for malaria diagnosis although it is not available in most peripheral health facilities. It is time consuming, requires trained personnel and needs careful preparation and application of reagents to ensure quality results. This study was aimed at testing the diagnostic performance of CareStart™ malaria rapid diagnostic test (RDT) with reference to light microscopy for the diagnosis of falciparum and vivax malaria in Ethiopia.

**Methods:**

Blood samples were collected from 254 patients suspected to have malaria at Kola Diba Health Center in the late malaria transmission peak season from November 2011 to December 2011. The samples were examined immediately by light microscopy and the RDT (CareStart™ Malaria HRP2/pLDH COMBO Test kit). Statistical analysis was performed using SPSS version 16 and the JavaStat two-way contingency table analysis.

**Results:**

The overall sensitivity and specificity of CareStart^TM^ RDT was found to be 95% (90–97.9%, 95% CI) and 94.2% (90.9–96%, 95% CI), respectively. The sensitivity of the CareStart^TM^ RDT for *Plasmodium falciparum* or mixed infection was calculated to be 92.9% (82.5–98%, 95%CI) while a sensitivity of 90.9% (74.1–98.4%, 95%CI) was found for non-*falciparum* species. The specificity for *P. falciparum* or mixed infections was found to be 95.4% (92.5–96.8%, 95%CI) while it was 97.3% (94.8–98.4%, 95%CI) for non-*falciparum* species. There was an excellent agreement between the two tests with a kappa value of 0.918.

**Conclusion:**

The CareStart^TM^ RDT test showed good sensitivity and specificity with an excellent agreement to the reference light microscopy. The RDT could therefore be used in place of light microscopy, which in poor set-ups cannot be used routinely.

## Background

Malaria is ranked as the leading communicable disease in Ethiopia, accounting for about 30% of the overall Disability Adjusted Life Years lost. Approximately 68% of the total population of 78 million lives in areas at risk of malaria [1]. According to Ethiopia’s Federal Ministry of Health (FMOH), in 2008/2009, malaria was the leading cause of outpatient visits, health facility admissions and inpatient deaths, accounting for 12% of reported outpatient visits and nearly 10% of admissions. Because a large proportion of the population does not have access to health care services, these figures probably under-estimate the true burden of malaria in the country [1]. In Ethiopia, *Plasmodium falciparum* and *Plasmodium vivax* are the major parasites accounting for about 70% and 30% of infections, respectively, during peak transmission periods [2,3].

The malaria transmission pattern in Ethiopia is highly seasonal and unstable [4]. Because of this unstable transmission and infrequent exposure to infection, immunity is generally under-developed and all age groups are at risk of malarial disease. Although pregnant women and children under five years of age are the most vulnerable groups, the population at age five and older is also high risk [4].

In the prevention and control of malaria, prompt and accurate diagnosis is the key to effective disease management [5]. However, in Ethiopia, clinical diagnosis and empirical treatment has been the mainstay of malaria management in areas where laboratory facilities are not available. Due to the non-specific nature of signs and symptoms of malaria, clinical diagnosis is unreliable [3,5,6]. In many countries malaria is still being diagnosed clinically, an unreliable method leading to over-diagnosis and over-treatment [7]. Light microscopy (LM) remains preferred and standard for laboratory diagnosis of malaria although it is not accessible and affordable in most peripheral health facilities in the country. Moreover, microscopy is time consuming, requires trained personnel and needs careful preparation and application of reagents to ensure quality results [6,8]. For a better and sustainable control, malaria diagnosis requires a more rapid, easy, sensitive and specific method.

Malaria rapid diagnostic test (RDT) was introduced in the 1990s and has undergone many improvements [9]. The CareStart™ Malaria HRP-2/ pLDH (Pf/PAN) Combo Test is a three-band RDT detecting HRP-2 and PAN-pLDH. RDTs for detection of *P. falciparum* and other species have been used by health extension workers (HEWs) at health posts in Ethiopia since 2005 [10-14]. It is a new tool for the rapid qualitative determination of malaria histidine-rich protein 2 (HRP2) and lactate dehydrogenase in human blood, aiding the diagnosis of malaria infection.

This study was conducted to evaluate the sensitivity and specificity of CareStart™ rapid malaria test in reference to the conventional LM in north-west Ethiopia.

## Methods

### Study area and period

The cross-sectional study was conducted at Kola Diba Health Center in Dembia District from November to December 2011, which was a peak transmission season in Ethiopia. The district is malarious and covers an area of 1,270 km^2^. The altitude of the district ranges between 1,750 and 2,100 m above sea level. It has a population of more than 300,000 and the majority of its population depends on subsistence farming. Kola Diba Health Center is one of the health centres in the district where people with symptoms suggestive of malaria obtain free services for diagnosis and treatment. Despite the presence of different control activities in the area, it remained endemic for both *P. vivax* and *P. falciparum* malaria despite a strong control effort.

### Study subjects

Two hundred and fifty four malaria suspected patients attending the medical and paediatric out-patient departments of Kola Diba Health Center during the study period were included and screened for malaria infection using LM and the CareStart™ RDT. Patients who had received anti-malarial drugs during the past four weeks and critically ill patients who were unable to give blood were excluded from the study.

### Specimen collection and processing

The socio-demographic characteristics and clinical data of the participants were collected using a structured and pre-tested questionnaire. Finger-prick samples were collected and placed in a grease-free, clean, glass slide. The same finger-prick blood sample was used to carry out the RDT in parallel, following manufacturer’s instructions. In a single slide, both thick and thin films were prepared. The thin films were fixed in methanol after air-drying, the slides were stained in 10% Giemsa solution for 15 min. Thin and thick films were read at the health centre by an experienced laboratory technician and the result was considered negative if no parasites were seen after examination of 200 fields at 1,000x magnification. The technician was blinded to the results of the RDT. All blood films were re-read a second time by an experienced microscopist at Gondar University Hospital laboratory who was also blinded to initial microscopy and RDT results. In cases where the results were discordant, a third expert reader was used. The results of the third expert reader were considered final. All the microscopists were from Gondar University Hospital laboratory. The laboratory is labelled as three star laboratory during the national pre-accreditation assessment by the Ethiopian Health and Nutrition Research Institute (EHNRI) which is on the final stages of accreditation by the WHO-AFRO. The laboratory is involved in external quality assurance (EQA) at EHNRI and the Amhara Regional Research and Diagnostic Laboratory. All the microscopists involved in the study had pre-study training and were qualified.

### Statistical analysis

The collected data were computerized using Excel program, exported and analysed by SPSS version 16 and JavaStat two-way contingency table analysis. Sensitivity, specificity, and positive and negative predictive values were determined for both tests and compared with one another. Kappa value was determined to see the consistency of the results among the diagnostic tools. A *P* value of less than 0.05 was considered significant in all comparisons.

### Ethical approval

Ethical clearance was obtained from the Institutional Review Board (IRB) of the University of Gondar, College of Medicine and Health Sciences. Verbal consent was taken from all study participants and mothers/guardians of all children under 18 after explaining the purpose and objective of the study. Participants who were diagnosed to have malaria were given the appropriate treatment at the health centre.

## Results

A total of 254 malaria-suspected patients were included in the study. The male to female ratio was 1.57:1 while the mean age (SD, range) of the participants was 21.4 years (14.76, 0.4–75). Most of the participants were from rural areas of the district (77.2%). Table 1 describes parasite positivity as detected by LM among different socio-demographic factors of patients. Using logistic regression analysis the sex (OR=0.551, 95%CI=0.325–0.932) and resident of the participants (OR=0.483, 95%CI=0.254–0.919) were found to be significantly associated with parasite positivity. Table 2 describes the presenting symptoms of patients and the rate of parasite positivity. Fever was the most commonly reported presenting symptom by the participants (99.2%) while headache (89.8%), sweating/chills/rigors (79.1%), fatigue (78.7%), and vomiting (50%) were other common presenting features. Only 40.9% (95%CI, 40.2–41.7%) of the febrile patients were diagnosed to have malaria. Using the logistic regression analysis it is only fatigue (OR=0.48, 95%CI=0.249–0.926) and splenomegally (OR=0.405, 95%CI=0.181–0.905) associated with malaria positivity using the LM.

**Table 1:** Parasite positivity as detected by light microscopy among different socio-demographic factors of patients using logistic regression analysis

**Patient variables**	**Parasite positivity by the LM**					
		**No. tested%**	**No. positive%**	**No. negative%**	**OR**	**95%CI**
Sex	Male	155(61)	72(46.5)	83(53.5)	0.551	0.325-0.932
	Female	99(39)	32(32.3)	67(67.7)		
Age group in years	<5	44 (17.3)	12(27.3)	32(72.6)	1.939	0.629-5.984
	6-15	46(18.1)	27(58.7)	19(41.3)	0.512	0.173-1.512
	16-25	84(33.1)	35(41.7)	49(59.3)	1.018	0.371-2.792
	26-35	44(17.3)	16(36.4)	28(63.6)	1.273	0.424-3.818
	36-45	17(6.7)	6(35.3)	11(64.7)	1.333	0.346-5.136
	>46	19(7.5)	8(42.1)	11(64.7)		
Residence	Rural	197(77.2)	88(44.9)	109(55.3)	0.483	0.254-0.919
	Urban	57(22.4)	16(28.1)	41(71.9)		
Educational status	Illiterate	155(61)	59(38.1)	96(61.9)	0.814	0.196-3.377
	Primary school	47(18.5)	29(61.7)	18(38.3)	0.31	0.069-1.399
	Secondary school	43(17)	13(30.2)	30(69.8)	1.154	0.25-5.335
	College	9(3.5)	3(33.3)	6(66.7)		
Occupation	Student	73(28.7)	31(42.5)	42(57.5)	1.185	0.389-3.617
	Farmer	141(55.5)	61(43.5)	80(56.7)		
	**Housewife**	25(9.8)	5(20)	20(80)	1.148	0.395-3.338
	**Others**	15(5.9)	7(46.7)	8(53.3)	3.50	0.854-14.342

CI=confidence interval; LM=light microscopy; OR=odds ratio.

**Table 2:** Parasite positivity detected by light microscopy based on patient signs and symptoms using logistic regression analysis

**Symptoms**	**Light microscopy**			
	**P**	**N**	**OR**	**95%CI**
Fever	103	149	1.447	0.89-23.39
Headache	97	131	0.498	0.201-1.231
Fatigue	89	111	0.48	0.249-0.926
Sweating/chills/rigours	87	114	0.619	0.326-1.174
Vomiting	54	73	0.878	0.532-1.448
Splenomegaly	17	11	0.405	0.181-0.905
Myalgia and arthralgia	18	24	0.910	0.466-1.778
Anaemia	5	6	0.825	0.245-2.778
Hypoglycaemia	6	7	0.8	0.261-2.451

CI=confidence interval; LM=light microscopy; N=negative; OR=odds ratio; P=positive.

The overall parasite positivity using LM was 104 (40.9%): 40 (15.7%) for *P. falciparum*, 58 (22.8%) for *P. vivax* and six (2.4%) for mixed infections. Using the CareStart^TM^ RDT, the overall parasite positivity was 100 (39.4%): 50 (19.7%) for *P. falciparum*, 24 (9.5%) for *P. vivax* and 26 (10.2%) for mixed infections (Tables 3). Difference in detection of malaria parasites using either the LM or the RDT was insignificant (*P*<0.001). The difference of in detection of *P. falciparum* parasite using either tests was also insignificant (*P*<0.001). However, difference in detection of *P. vivax* using the LM and RDT was found to be significant (*P*<0.001). Considering mixed infection as *P. falciparum*, the difference of detection was found to be significant (*P*<0.001).

**Table 3:** **Malaria parasite positivity based on light microscopy and CareStart**^**TM**^**RDT**

**LM**	**CareStart**^**TM**^**RDT**					
	***P.falciparum*Number (%)**	***P. vivax*(PAN only) Number (%)**	**Mixed (pf+PAN) Number (%)**	**Negative Number (%)**	**Total Number (%)**	P value*
***P.falciparum***	18 (7.1)	2 (0.8)	15 (5.9)	5 (1.9)	40 (15.7)	<0.001
***P. vivax***	28 (11)	20(7.9)	6(2.4)	4(1.6)	58(22.8)	
**Mixed**	1(0.4)	0 (0)	5(1.9)	0(0)	6(2.4)	
**Negative**	3(1.2)	2(0.8)	0(0)	145(57.1)	150(59.1)	
**Total**	50 (19.7)	24 (9.4)	26(10.2)	154(60.6)	254(100)	

LM=light microscopy; Pf=*Plasmodium falciparum*; RDT=rapid diagnostic test.

* Chi square test was used to calculate the p value.

Taking the LM as a standard test for malaria, the sensitivity and specificity of CareStart^TM^ RDT was found to be 95% (90–97.9%, 95% CI) and 94.2% (90.9–96%, 95% CI), respectively. The positive predictive value (PPV) and the negative predictive value (NPV) were found to be 91.3% (86.5–94.1%, 95% CI) and 96.7% (93.3–98.6%, 95% CI), respectively. There was an excellent agreement between the LM and CareStart^TM^ RDT with a Kappa value of 0.918 (0.852–0.955, 95% CI) (Table 4).

**Table 4:** **Performance characteristics of CareStart**^**TM**^by species identified in comparison to the standard reference light microscopy*

**Test**	**Sensitivity% (95% CI)**	**Specificity% (95% CI)**	**PPV% (95% CI)**	**NPV% (95% CI)**	**Agreement between tests Kappa (95% CI)**
Pf or mixed by LM *vs* pf/pan by RDT	92.9 (82.5-98)	95.4 (92.5-96.8)	84.8(75.3-89.5)	98 (95–99.4)	0.853(0.726-0.916)
Non-Pf (by LM) *vs* Pan only (by RDT)	90.9 (74.1-98.4)	97.3 (94.8-98.4)	83.3 (67.9-90.1)	98.6 (96.1-99.7)	0.849(0.663-0.931)
All positive by LM *vs* all positive by RDT	95 (90–97.9)	94.2 (90.9-96)	91.3( 86.5-94.1)	96.7(93.3-98.6)	0.885(0.803-0.933)

Pf=*Plasmodium falciparum*; LM=light microscopy; RDT=rapid diagnostic test; PPV=positive predictive value; NPV=negative predictive value.

*P<0.001.

Since both the Pf-specific and the ‘PAN’ antigen used by the CareStart^TM^ RDT are detected in *P. falciparum* infections, giving the result “Pf plus PAN” even for non-mixed *P. falciparum* infections, it was not possible to directly compare the performance of the test against *P. falciparum* single infections detected by LM. Therefore, the sensitivity and specificity of “Pf or mixed” (by LM) to “Pf plus PAN” (by RDT) were compared, whilst non-*falciparum* infections detected by LM were compared directly to the “PAN only” RDT results (Table 4). Accordingly, the sensitivity of the CareStart^TM^ RDT for *P. falciparum* or mixed infection compared to LM was calculated to be 92.9% (82.5–98%, 95%CI) while a sensitivity of 90.9% (74.1–98.4%, 95%CI) was found for non- *falciparum* species. The specificity for *P. falciparum* or mixed infections was found to be 95.4% (92.5–96.8%, 95%CI) while it was 97.3% (94.8–98.4%, 95%CI) for non*-falciparum* species (Table 4).

Of note in the present study was an excellent agreement between CareStart^TM^ RDT and LM with a Kappa value of 0.918. There was also a very good agreement between the RDT and LM in detecting different species of *Plasmodium*: Kappa value of 0.853 for *P. falciparum* or mixed infection and Kappa value of 0.849 for non-*falciparum* species.

## Discussion

The present study revealed a high sensitivity and specificity of the CareStart^TM^ RDT (Table 4). The high sensitivity of the RDT in this study was in line with other studies from south-west Ethiopia [11] and Madagascar [15]. The current study revealed a higher sensitivity and a slightly lower specificity than reports from Myanmar [16]. Overall, the CareStart^TM^ RDT showed good sensitivity when compared to the LM. In set ups where health personnel rely on their clinical judgment, using RDT for the diagnosis of malaria can be helpful for early institution of treatment.

This study had also tried to evaluate the performance of the CareStart^TM^ RDT in detecting different species of malaria parasite. The finding in the current study was higher than that reported by Ashton *et al* from Oromia Regional State of Ethiopia [17]. The sensitivity of the RDT in the current study for *P. falciparum* or mixed infection was also higher than that by Maltha *et al* (78.5%) [18]. However, the sensitivity in this study was found to be lower than reports from south-west Ethiopia [11], south Ethiopia [12] and Madagascar [15]. These differences could be due to observer variation, difference with malaria species circulating at different localities or host factors [19].

The specificity of the CareStart^TM^ in the present study for *P. falciparum* or mixed infections was higher than the reports from Oromia Regional State in Ethiopia and Madagascar [15,17] and lower than the reports from southern [12] and south-west Ethiopia [11]. The specificity for the non-*falciparum* species in the current study was comparable to the reports of some studies [17], higher in some others [15] and lower in elsewhere [11,12]. The differences in the specificity of the RDT could be due to the aforementioned reasons [19].

The RDT had high NPV, meaning that it was reliable in ruling out malaria. Similarly, the higher PPV means that patients will be correctly diagnosed as positive for malaria and avoids unnecessary treatment.

The overall prevalence of malaria in the study area was very high, as detected by either the CareStart^TM^ RDT (39.4%) or the LM (40.9%). The result was higher than the report from Oromia Regional State in Ethiopia (23.2%) [17] while it was in agreement with a report from three regions in Ethiopia [20]. The high prevalence could be partly explained by the fact that the study was conducted in a peak malaria transmission season in the country. Also, the malaria transmission pattern in Ethiopia is highly seasonal and unstable. Because of this unstable transmission and infrequent exposure to infection, immunity is generally under-developed and all age groups are at risk of malarial disease [19]. On the other hand, it might be due to development of anti-malarial or insecticide resistance in the area [5]. The knowledge, attitude and practice of the participants could also be a factor [19]. However, these assumptions should be evaluated with further studies. The high prevalence of malaria, despite the tremendous effort to distribute bed nets and apply outdoor insecticides, heralds the need to evaluate the malaria control system in the area and beyond.

Current subjective or objective fever (axillary temperature of >37.5^0^c) was the most common presenting symptom by the participants (Table 2). Fever detects only 40.9% malaria. This could be explained by the fact that individuals may carry parasites without symptoms. On the other hand, the significant overlap of malaria symptoms with other tropical diseases might have impaired the specificity of fever and encouraged the indiscriminate use of anti-malarials for managing febrile conditions in endemic areas. Studies of fever cases in Philippines, Sri Lanka, Thailand, Mali, Chad, Tanzania and Kenya have shown high percentages of malaria over-diagnosis when using fever as a clinical diagnostic tool [21-27]. Comprehensive investigation to identify the etiologic agents of febrile illnesses could be helpful in the study area and beyond. Defining the malaria-attributable fraction to estimate the frequency of true febrile malaria among all febrile cases, by fitting the risk of fever as a function of parasite density using a logistic regression model, would be of paramount importance [28].

## Conclusion

The CareStart^TM^ RDT test showed good sensitivity and specificity with an excellent agreement to the reference LM. The RDT could therefore be used in place of LM, which in poor set-ups cannot be used routinely.

## Abbreviations

CI, Confidence Interval; HEWs, Health Extension Workers; HRP-2, Histidine-Rich Protein 2; IRB, Institutional Review Board; LDH, Lactate Dehydrogenase; LM, Light Microscopy; NPV, Negative Predictive Value; OR, Odds Ratio; PPV, Positive Predictive Value; RDT, Rapid Diagnostic Test; WHO, World Health Organization.

## Competing interests

The authors declare that they have no competing interests.

## Authors’ contribution

BM was involved in the study conception and design, data analysis, and drafting the manuscript. AK, AM BA, YB and ZT were involved in study conception and design and drafting the manuscript. MG, AG and DW were involved in data collection and reviewing the manuscript. All the authors have read, edited and approved the manuscript.
